# The serine-48 residue of nucleolar phosphoprotein nucleophosmin-1 plays critical role in subcellular localization and interaction with porcine circovirus type 3 capsid protein

**DOI:** 10.1186/s13567-020-00876-9

**Published:** 2021-01-07

**Authors:** Jianwei Zhou, Juan Li, Haimin Li, Ying Zhang, Weiren Dong, Yulan Jin, Yan Yan, Jinyan Gu, Jiyong Zhou

**Affiliations:** 1 MOA Key Laboratory of Animal Virology, Center of Veterinary Sciences, Zhejiang University, 866 Yuhangtang Road, Hangzhou, Zhejiang 310058 PR China; 2 Collaborative Innovation Center and State Key Laboratory for Diagnosis and Treatment of Infectious Diseases, First Affiliated Hospital, Zhejiang University, Hangzhou, PR China

**Keywords:** porcine circovirus type 3, capsid protein, nucleolar localization signal, nucleolar phosphoprotein nucleophosmin-1, amino acid charge property

## Abstract

The transport of circovirus capsid protein into nucleus is essential for viral replication in infected cell. However, the role of nucleolar shuttle proteins during porcine circovirus 3 capsid protein (PCV3 Cap) import is still not understood. Here, we report a previously unidentified nucleolar localization signal (NoLS) of PCV3 Cap, which hijacks the nucleolar phosphoprotein nucleophosmin-1 (NPM1) to facilitate nucleolar localization of PCV3 Cap. The NoLS of PCV3 Cap and serine-48 residue of N-terminal oligomerization domain of NPM1 are essential for PCV3 Cap/NPM1 interaction. In addition, charge property of serine-48 residue of NPM1 is critical for nucleolar localization and interaction with PCV3 Cap. Taken together, our findings demonstrate for the first time that NPM1 interacts with PCV3 Cap and is responsible for its nucleolar localization.

## Introduction

Viruses of genus *Circovirus*, in the family *Circoviridae*, have been detected in terrestrial, aquatic and avian species, including pigs, ducks, dogs, minks, rats, palm civets, geese, pigeons, canaries, parrots, and others [1–8]. Circoviruses are circular, single-stranded DNA viruses that are the smallest known autonomously replicating viruses in mammals [9]. Two porcine circovirus (PCV) genotypes, PCV1 and PCV2, have been extensively studied [10, 11]. PCV1 is nonpathogenic to pigs and was identified in the porcine kidney cell line PK-15 [12, 13]. PCV2 is pathogenic and causes porcine circovirus-associated diseases (PCVAD) and immunosuppression [12, 14, 15].

In 2015, a novel PCV, designated as PCV3, was first identified by next generation sequence (NGS) analysis as a pathogenic agent in sows that died and displayed acute porcine dermatitis and nephropathy syndrome (PDNS)-like clinical signs, reproductive failure, cardiac pathology, and multisystemic inflammation in the United States [15, 16] and then in China, Poland, South Korea, Brazil, Thailand, Germany, Denmark, Spain, and Italy [17–24]. Retrospective research data have shown that PCV3 possesses high sequence homology to bat circovirus and that the earliest cases of PCV3 infection can be traced back to 1966 in China, suggesting that PCV3 may have been derived early from bats and gradually adapted to pigs [25]. Recently, Fu et al. found that PCV3 has been circulated in swine herds for nearly half a century and may have originated from a bat-associated circovirus [26]. This implicates that in addition to PCV2, the newly discovered PCV3, is also pathogenic to pigs and serves as an etiological agent for PCVAD.

The PCV3 genome is a single-stranded circular DNA of 2.0 kb, which contains three major open reading frames (ORFs) [16]. The ORF2-encoded capsid protein (Cap) is necessary for virion packaging and participates in genome replication by interacting with Rep protein and is also the main viral immunogen and acts as a key regulator of viral replication as well as the virus-host interaction [27–34]. The N-terminus of PCV3 Cap is rich in basic amino acids, and the amino acid residues 1–34 was identified as nuclear localization signal (NLS), which is essential for nuclear transport of PCV3 Cap [35]. Besides, PCV3 Cap interacts with signal transducer and activator of transcription 2 (STAT2) to inhibit type I interferon signaling [36]. Nuclear entry of viral proteins is responsible for genome replication and is mediated by cellular shuttle proteins. The capsid of herpes simplex virus type 1 (HSV-1) and adenovirus 2 (AdV-2), the nucleoprotein of influenza A virus (IAV), and NS5A of hepatitis C virus (HCV) bound to nuclear pore complex (NPC) proteins, nuclear import soluble factors, heat shock protein 70 (HSP70), and histone H1 for nuclear shuttling, which were essential for viral propagation [37–39]. Besides NLS, there may be involvement of host proteins for nuclear entry of PCV3 Cap. For example, hepatitis delta virus (HDV) was studied for nucleolar localization and it was found that interacting with nucleolin (NCL) promoted viral replication [40]. Since PCV3 Cap is a nucleus-located protein, whether host proteins participating in its localization need to be elucidated.

Nucleolar phosphoprotein nucleophosmin-1 (NPM1) is a nuclear shuttle factor and resides mainly in the nucleolus, which bears a N-terminal oligomerization domain (OligoD), a central histone-binding domain (HistonD), and a C-terminal nucleic acid-binding domain (NBD) [41, 42]. NPM1 plays roles in many cellular processes such as ribosome biogenesis, DNA replication and repair, stress response, centrosome duplication, and nucleo**–**cytoplasmic transport [43]. NPM1 has been reported to interact with viral proteins, such as human immunodeficiency virus type 1 (HIV-1) Rev protein [44] and adenoviral core proteins [45]. However, whether NPM1 interacts PCV3 Cap remains unclear.

In the present study, we found that knockdown of *NPM1* abolished nucleolar localization of PCV3 Cap, and confirmed the essential role of NoLS of PCV3 Cap for binding to NPM1. We proved that charge property of serine-48 residue within N-terminal OligoD of NPM1 determines its subcellular localization and interaction with PCV3 Cap. Taken together, our findings highlight the critical role of NPM1 in nucleolar entry of PCV3 Cap.

## Materials and methods

### Cells

The Epithelial PK-15 cell line, free of PCV was provided by the China Institute of Veterinary Drugs Control and was maintained in our laboratory. The cell line was cultured in minimal essential medium (MEM) (Gibco, Carlsbad, CA, USA). HEK293T cells (CRL-11268; ATCC, Manassas, VA, USA) were cultured in Dulbecco’s modified Eagle medium (DMEM) (Gibco). Both media were supplemented with 10% fetal bovine serum (FBS) (CCS30010.02; MRC, Australia).

### Antibodies and reagents

Rabbit polyclonal antibodies (pAb) against Myc (R1208-1), GFP (SR48-02), Flag (0912-1), and mouse monoclonal antibodies (mAb) against β-actin (M1210-2) and GST (M0807-1) were purchased from Huaan Biological Technology (Hangzhou, China). Mouse anti-Myc (05-419) and anti-Flag (F1804) mAbs were obtained from Sigma-Aldrich (St. Louis, MO, USA). Anti-Flag affinity resin (A2220) for immunoprecipitation was also purchased from Sigma-Aldrich. Rabbit mAb against NPM1 (ab52644) was purchased from Abcam (Cambridge, MA). NP-40 cell lysis buffer (50 mM Tris [pH 7.4], 150 mM NaCl, 1% NP-40) was purchased from Beyotime (P0013F; Shanghai, China). Horseradish peroxidase (HRP)-labeled goat anti-mouse and anti-rabbit IgG were purchased from KPL (Milford, MA, USA).

### Plasmid construction and transfection

DNA fragments encoding full-length and truncated PCV3 *Cap* variants were amplified by PCR from full-length genomic DNA of PCV3 [16] (accession no. KT869077.1), and subcloned separately into vectors pCMV-Myc-N (Clontech, Palo Alto, CA, USA), pCMV-Flag-N (Clontech), pCMV- Flag-gst-N (Clontech), pEGFP-C3 (Clontech), and pmCherry-C1 (Clontech) for different purposes. The resultant plasmids were Flag-PCV3-Cap, Flag-gst-PCV3-Cap, Myc-PCV3-Cap, GFP-PCV3-Cap, GFP-PCV3-Cap(1-34aa), GFP-PCV3-Cap(35-214aa), mCherry-PCV3-Cap, mCherry-PCV3-Cap(1-34aa), mCherry-PCV3-Cap(35-214aa). The nucleotide fragments PCV1-NLS, PCV4-NLS, CanaryCV-NLS, CanineCV-NLS, MinkCV-NLS, DragonflyCV-NLS, CoCV-NLS, BtCV-NLS, DuCV-NLS, GoCV-NLS, and BFDV-NLS were synthesized and inserted into the vector pEGFP-C3 by Sunya Biotechnology (Zhejiang, China). GFP-PCV2-NLS plasmid was constructed and stored in our laboratory. The nuclear localization signals (NLSs) within capsid protein of circoviruses from different species are listed in Table 1. The full-length cDNA sequences of *NCL* (accession no. XM_021074959.1), *NPM1* (accession no. XM_013990662.2), and truncated *NPM1* variants were amplified from PK-15 cells and cloned into vectors pmCherry-C1, pCMV-Flag-N, pEGFP-C3, and pGEX-4T-1 (GE Healthcare Biosciences, Piscataway, NJ, USA) using specific primers. The resultant plasmids were mCherry-NCL, GFP-NCL, mCherry-NPM1, Flag-NPM1, GST-NPM1, GFP-NPM1(1-294aa), GFP-NPM1(1-117aa), GFP-NPM1(118-188aa), GFP-NPM1(189-294aa), GFP-NPM1(1-188aa), GFP-NPM1(118-294aa), and GFP-NPM1(1-117aa + 189-294aa). Mutants were generated by site-specific mutagenesis. The primers used are summarized in Table 2. For transfection, PK-15 and HEK293T cells were seeded onto plates or glass coverslips at a suitable density according to the experimental plan and grown up to 70% to 90% confluency. jetPRIME transfection reagent (Polyplus Transfection, New York, NY, USA) was used for PK-15 cell transfection, and ExFect transfection reagent (T101-01/02; Vazyme Biotechnology, Nanjing, China) was used for 293T cell transfection according to the manufacturers’ instructions.

**Table 1:** **The nuclear localization signals (NLSs) of circovirus Cap from different species**

Circoviruses	Accession No	Sequences of NLSs
PCV1	AY193712.1	MTWPRRRYRRRRTRPRSHLGNILRRRPYLAHPAFRNRYRWRRK
PCV2	AY188355.1	MTYPRRRYRRRRHRPRSHLGQILRRRPWLVHPRHRYRWRRK
PCV3	KT869077.1	MRHRAIFRRRPRPRRRRRHRRRYARRRLFIRRPT
PCV4	MK986820.1	MPIRSRYSRRRRNRRNQRRRGLWPRANRRRYRWRRKN
CanaryCV	AJ301633.2	MWLTFNQVARRRRPLAPRRRRWRRRYWXRRRRIPANRR
CanineCV	MK731981.1	MRVRRHARASRRRYRTRPLIRYRRRRQNNFKLFHLRLRR
MinkCV	MK561562.1	MPVRSRYSRRRRWRRNRRRRGPQRRYARGGYRWRR
DragonflyCV	HQ638058.1	MVRYRRAARRPVRRARRSRVRKLRFRRRRRYHRR
CoCV	HQ401281.1	MRRRRFYRRRRAPIRRRRIRRRRTRLSRMRRGHR
BtCV	JF938082.1	MARFRRRVARRRPVRSIRRIRRRRRYGRRR
DuCV	EF370476.1	MRRSTYRRAYRARRRRRGLRRRLRRRRLRIGRPRRR
GoCV	AF536940.1	MPLYRARPRSLYYRRRRAANRRRRYRRRRLHIGRIR
BFDV	AY450445.1	MNCACAIFQIRRRRYARPYYRRRHNRRYRRRRRYFRRRR

**Table 2:** **Primers used for cloning PCR**

Gene product	Sense primer (5′–3′)	Antisense primer (5′–3′)
PCV3 Cap(1-214aa)	ATGAGACACAGAGCTATATTC	TTAGAGAACGGACTTGTAACGAATC
PCV3 Cap(35-214aa)	ATGGCTGGCACATACTACACAAAGAAAT	TTAGAGAACGGACTTGTAACGAATC
PCV3 Cap(1-34aa)	ATGAGACACAGAGCTATATTC	TTA TGTGGGCCTCCTAATGAATA
NPM1(1-294aa)	ATGGAAGATTCGATGGATAT	TTAAAGAGACTTCCTCCACT
NPM1(1-117aa)	ATGGAAGATTCGATGGATAT	TACTAAGTGCTGTCCACTAATATGC
NPM1(118-188aa)	GCTGTAGAGGAAGATGCAGAGT	TTCCGCTTCCTCATCATCAAA
NPM1(189-294aa)	GAAAAAGCTCCAGTAAAGAAAT	TTAAAGAGACTTCCTCCACT
NPM1(1-188aa)	ATGGAAGATTCGATGGATAT	TTCCGCTTCCTCATCATCAAA
NPM1(118-294aa)	GCTGTAGAGGAAGATGCAGAGT	TTAAAGAGACTTCCTCCACT
NPM1(1-117aa + 189-294aa)	ATGGAAGATTCGATGGATAT	CTTTACTGGAGCTTTTTCTACTAAGTGCTGTCCACTA
	TAGTGGACAGCACTTAGTAGAAAAAGCTCCAGTAAAG	TTAAAGAGACTTCCTCCACT
	ATGGAAGATTCGATGGATAT	TTAAAGAGACTTCCTCCACT
NPM1-S48A	TTATCTTTAAGAACGGTCGCATTAGGGGCT	AGCCCCTAATGCGACCGTTCTTAAAGATAA
NPM1-S48E	TTATCTTTAAGAACGGTCGAATTAGGGGCTGGCGCAAA	TTTGCGCCAGCCCCTAATTCGACCGTTCTTAAAGATAA
NPM1-S88A	AACTTTGAAAATGTCTGTACAGCCGACGGTTGCACTTG	CAAGTGCAACCGTCGGCTGTACAGACATTTTCAAAGTT
NPM1-S88E	AACTTTGAAAATGTCTGTACAGCCGACGGTTGAACTTG	CAAGTTCAACCGTCGGCTGTACAGACATTTTCAAAGTT
NPM1-T95A	TTGGGGGCTTTGAAATAGCACCACCTGTGGTCTTA	TAAGACCACAGGTGGTGCTATTTCAAAGCCCCCAA
NPM1-T95D	TTGGGGGCTTTGAAATAGATCCACCTGTGGTCTTA	TAAGACCACAGGTGGATCTATTTCAAAGCCCCCAA
NPM1-S48T	TTATCTTTAAGAACGGTCACATTAGGGGCT	AGCCCCTAATGTGACCGTTCTTAAAGATAA
NPM1-S48D	AAAATGAGCACCAGTTATCTTTAAGAACGGTCGATTTA	TAAATCGACCGTTCTTAAAGATAACTGGTGCTCATTTT
NPM1-S48K	TTATCTTTAAGAACGGTCAAATTAGGGGCTGGCGCAAA	TTTGCGCCAGCCCCTAATTTGACCGTTCTTAAAGATAA
NPM1-S48R	TTATCTTTAAGAACGGTCAGATTAGGGGCTGGCGCAAA	TTTGCGCCAGCCCCTAATCTGACCGTTCTTAAAGATAA

### Confocal microscopy

To study the colocalization of NPM1, NCL and full-length PCV3 Cap or its truncated variants, HEK293T or PK-15 cells were cotransfected with indicated vectors fused with GFP and mCherry tags. The cells were fixed using 4% paraformaldehyde (PFA) for 20 min and permeabilized with 0.2% Triton X-100 for 5 min at room temperature. Cellular nuclei were counterstained with 10 μg/mL 4′, 6′-diamidino-2-phenylindole (DAPI; 10236276001; Roche, Mannheim, Germany). The cells were then visualized under LSM780 laser scanning confocal microscope (Zeiss, Oberkochen, Germany). GFP signal was detected after excitation at 488 nm with an emission long-band filter at 505–530 nm (green). mCherry fluorescence was detected after excitation at 561 nm with an emission long-pass filter at 550–600 nm (red). DAPI was detected after excitation at 405 nm with an emission long-pass filter at 445–450 nm (blue).

### SDS-PAGE and immunoblotting

For western blotting, cells were lysed in lysis buffer after transfection. Samples were collected, and proteins were separated by standard SDS-PAGE and transferred to nitrocellulose membranes (GE Healthcare) followed by blocking in phosphate-buffered saline (PBS) containing 5% skimmed milk powder and 0.05% Tween 20. The membrane was washed three times with PBS containing 0.05% Tween 20. After that, the membranes were incubated with primary antibody overnight at 4 °C. After three to five washes with PBS containing 0.05% Tween 20, the membranes were incubated with HRP-labeled secondary antibody at room temperature for 1.0 h. Immunoreactive protein bands were then visualized using enhanced chemiluminescence reagent (Amersham Biosciences, Little Chalfont, United Kingdom) and imaged using AI680 Images (GE Healthcare).

### Co-IP and GST pull-down assays

For Co-IP assays, HEK293T cells were transfected with the indicated plasmids for 48 h. Cells were lysed in NP-40 cell lysis buffer containing a protease inhibitor cocktail. After centrifugation at 12,000 × *g* for 10 min, the supernatant was treated with protein A/G plus agarose (sc-2002; Santa Cruz Biotechnology, Santa Cruz, CA, USA) for 1.0 h at 4 °C to eliminate nonspecific binding to the agarose beads, and then immunoprecipitated using anti-Flag beads. The beads were washed three times with NP-40 buffer and then boiled in protein loading buffer before the SDS-PAGE and western blotting. For GST pull-down assays, GST and GST-NPM1 were expressed in *Escherichia coli* BL21 cells and purified using Pierce glutathione agarose beads (21516; Thermo, Rockford, IL, USA). To prepare the bait proteins, purified GST as well as the GST-NPM1 were immobilized on glutathione agarose beads (16100; Thermo Fisher Scientific, USA), while lysates of Flag-PCV3-Cap transfected 293T cells were used as the prey protein. Equal amount of purified GST, GST-NPM1 and Flag-PCV3-Cap proteins were added together and incubated overnight at 4 °C and washed three times with NP-40 buffer. After that, it was boiled in protein loading buffer and subjected SDS-PAGE and western blotting using mouse mAbs against Flag and GST. For the unconventional GST pull-down assays, HEK293T cells were transfected with the indicated plasmids for 48 h. Cells were lysed in NP-40 buffer containing a protease inhibitor cocktail. Subsequently, the cell lysates were precleared and immobilized on glutathione agarose beads and incubated for 4.0 h at 4 °C. The beads were then washed three times with NP-40 buffer and boiled in protein loading buffer. Finally, the protein samples were separated and subjected to western blotting using mouse mAbs against Flag and GST or rabbit pAb against GFP.

### *NPM1* knockdown by lentivirus-mediated RNA interference (RNAi)

*NPM1* knockdown was performed as previously described [46] with slight modifications. Briefly, the effective shNPM1 (targeting sequence GGATGAGTTGCACATTGTATT) or shCON (targeting sequence CGGATCGCTACAAATAAG) RNA was co-transfected with the helper lentiviral packaging plasmids psPAX2.0 (12260; Addgene) and pMD2.G (12259; Addgene) into HEK293T cells to produce a lentivirus containing shNPM1 or shCON respectively. PK-15 cells were then infected with the resultant lentiviruses and cultured in complete medium for another 24 h and then subjected to puromycin (5 µg/mL; A1113803; Invitrogen) treatment for 7 days to obtain *NPM1*-silenced cells. Finally, the nuclear DNA content of shRNA-control and *NPM1*-silenced cells were measured using propidium iodide (PI) staining and fluorescence-activated cell sorting (FACS) analysis to analyze cell cycle.

### Cell cycle analysis

Flow cytometry was used to analyze cell cycle. In brief, the adherent shCON and shNPM1 cells were harvested by trypsin digestion, washed with PBS, and pelleted by centrifugation. Afterwards, the cells were fixed in 70% ethanol overnight at 4 °C and then stained for nuclear DNA content with 50 μg/mL FxCycle^TM^PI/RNase Staining Solution (2149269; Thermo Fisher Scientific, USA) at room temperature for 30 min. Determination of PI-stained cells was performed using fluorescence-activated cell sorting (FACS) on BD FACSverse. At least 100,000 cells were counted for each sample. Data were analyzed using FlowJo software.

### Statistical analysis

All results are presented as means ± standard deviations (SD). Statistical analysis was performed using Student’s *t-*test. *p-*values of < 0.05 were considered significant.

## Results

### The NPM1 is essential for the nucleolar localization of PCV3 Cap

**Figure 1:**
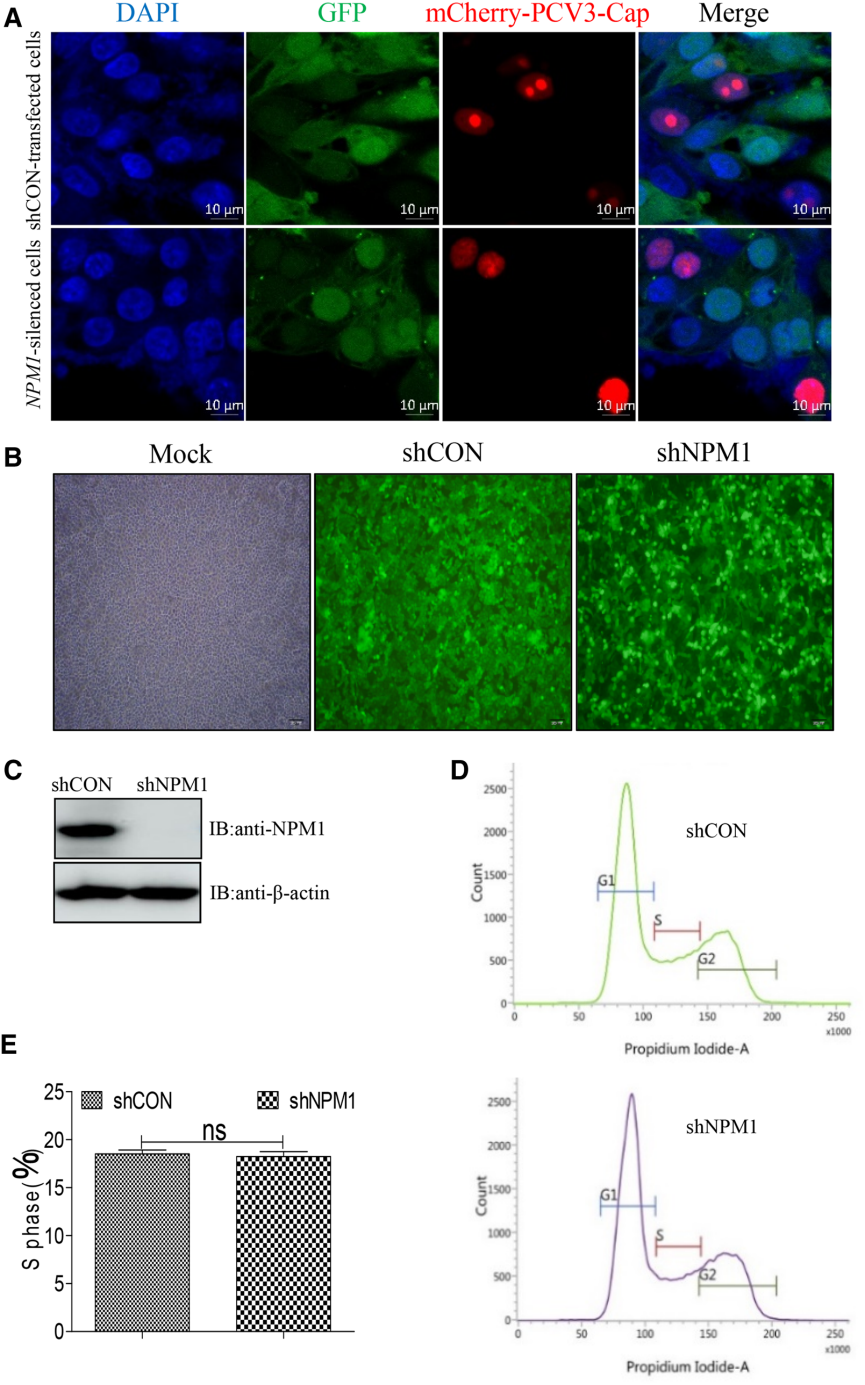
**NPM1 knockdown abolished the nucleolar localization of PCV3 Cap.** **A** *NPM1*-knocked down and shCON-transfected PK-15 cells were respectively transfected with mCherry-PCV3-Cap for 24 h, and cells were fixed and then subjected to confocal microscopy analysis. GFP signals indicate silenced PK-15 cells. Nuclei were stained with 4′, 6′-diamidino-2-phenylindole (DAPI). Scale bar, 10 μm. **B**, **C** Images of PK-15 cell line expressing shRNA mediated by lentivirus (**B**), and the knockdown efficiency of NPM1 in shCON-transfected and *NPM1*-silenced PK-15 cells (**C**). The cell lysates were subjected to immunoblotting using the indicated antibodies. **D** The nuclear DNA content of shRNA-control and *NPM1*-silenced cells were measured using PI staining and FACS analysis to analyze the cell cycle. **E** The histogram was analyzed quantitatively by a curve-fitting program to determine the percentage of cells in the S phase of both cells. Data are presented as means ± SD of three independent biological experiments. *ns* not significant.

To investigate the role of NPM1 in the nucleolar localization of PCV3 viral protein Cap, the *NPM1*-silenced and control PK-15 cells were transfected with mCherry-PCV3-Cap for 24 h. Confocal imaging showed that PCV3 Cap exhibited apparent nucleolar localization in shCON-transfected cells, and distributed to the nucleoplasm in *NPM1*-silenced cells (Figure 1A). The knockdown level of NPM1 in shCON-transfected and *NPM1*-silenced PK-15 cells was apparent as shown in Figure 1B, C. To rule out the effect of cell cycle (S phase) on the nucleolar localization of PCV3 Cap, the nuclear DNA content of shRNA-control and *NPM1*-silenced cells was measured using propidium iodide (PI) staining and fluorescence-activated cell sorting (FACS) analysis. The results indicated that the G0/G1, S, and G2/M phases in *NPM1*-silenced cells were nearly same as compared to that in the shRNA control cells (Figure 1D). The histogram was further analyzed quantitatively by a curve-fitting program to determine the percentage of cells in the S phase. The percentage was similar in both group of cells (Figure 1E). Taken together, the results indicate that the NPM1 is responsible for supporting the nucleolar localization of PCV3 Cap.

### PCV3 Cap colocalizes and interacts with nucleolar protein NPM1

**Figure 2:**
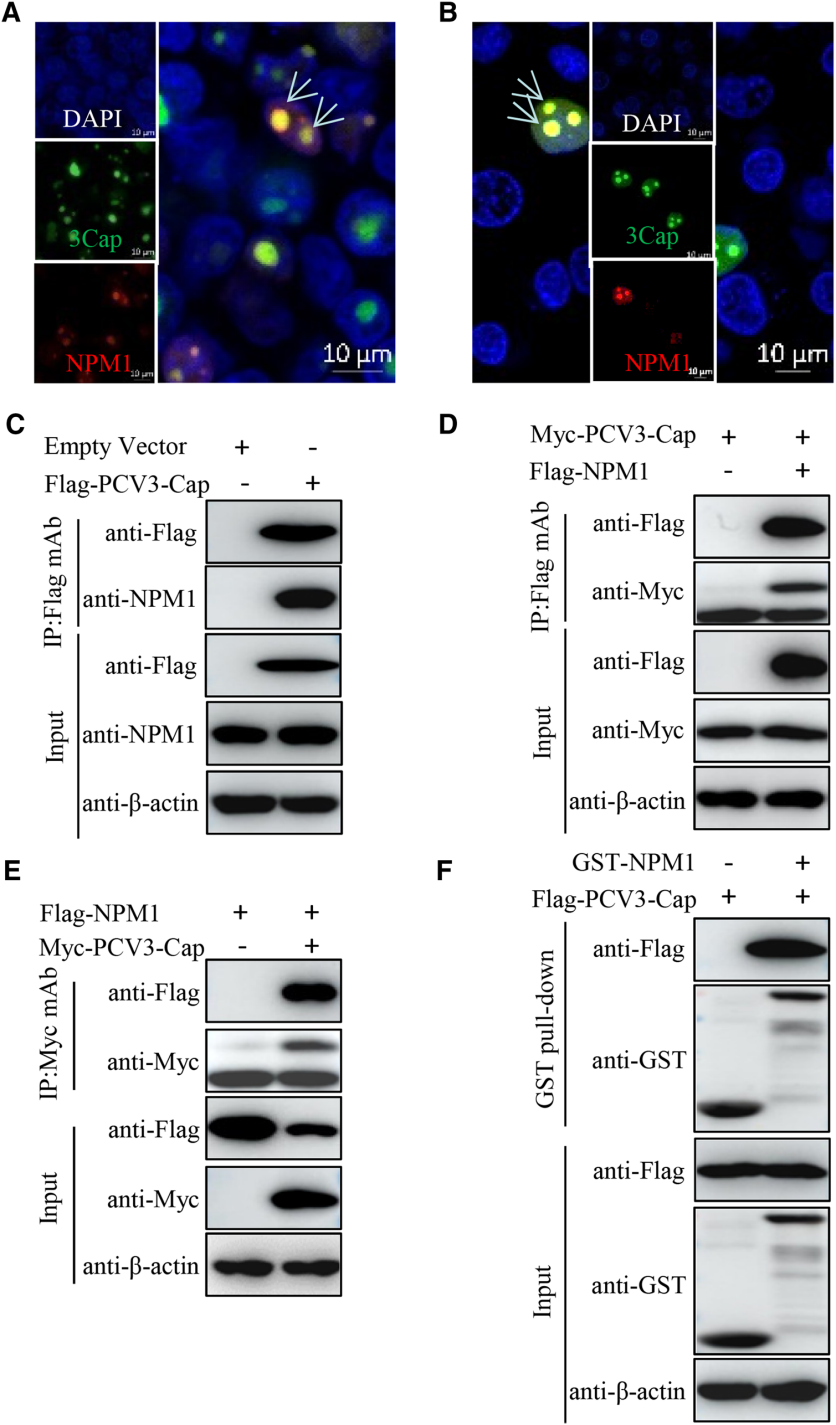
**PCV3 Cap colocalizes and interacts with NPM1.** **A**, **B** NPM1 colocalization with PCV3 Cap in transfected cells. HEK293T (**A**) and PK-15 (**B**) cells were cotransfected with GFP-PCV3-Cap and mCherry-NPM1 for 24 h, and cells were fixed and then subjected to confocal microscopy analysis. Nuclei were stained with 4′, 6′-diamidino-2-phenylindole (DAPI) (**A**, **B**). Scale bar, 10 μm. **C**. PK-15 cells were transfected with empty vector and Flag-PCV3-Cap for 48 h. **D**, **E** HEK293T cells were cotransfected with Myc-PCV3-Cap and Flag-NPM1 for 48 h. The cell lysates were immunoprecipitated with Flag beads (**C**, **D**) or anti-Myc purified IgG (**E**). **F** Lysates of Flag-PCV3-Cap transfected 293T cells mixed with the GST, GST-NPM1 proteins were pulled down with glutathione *S*-transferase (GST) beads, and then subjected to GST pull-down assays, followed by immunoblotting using corresponding antibodies.

Given that silencing NPM1 expression could repress the nucleolar localization of PCV3 Cap, we investigated the colocalization of PCV3 Cap with NPM1. The results showed that PCV3 Cap colocalized with NPM1 in nucleoli of both HEK293T and PK-15 cells cotransfected with GFP-PCV3-Cap and mCherry-NPM1 (Figure 2A, B). To confirm the interaction between PCV3 Cap and NPM1, lysates of PK-15 cells transfected with Flag-PCV3-Cap were immunoprecipitated with anti-Flag beads and probed for the presence of NPM1 protein with anti-NPM1 monoclonal antibody (mAb), indicating that PCV3 Cap indeed interacted with endogenous NPM1 protein (Figure 2C). Consistently, Flag-tagged NPM1 could interact with Myc-tagged PCV3 Cap in 293T cells (Figure 2D, E). Furthermore, glutathione *S*-transferase (GST) pull-down assays and immunoblotting revealed a direct interaction of NPM1 with PCV3 Cap (Figure 2F).

Taken together, these data indicated that PCV3 Cap interacts directly with NPM1.

### The N-terminal residues 1–34 of PCV3 Cap is a nucleolar localization signal and critical for binding to NPM1

**Figure 3:**
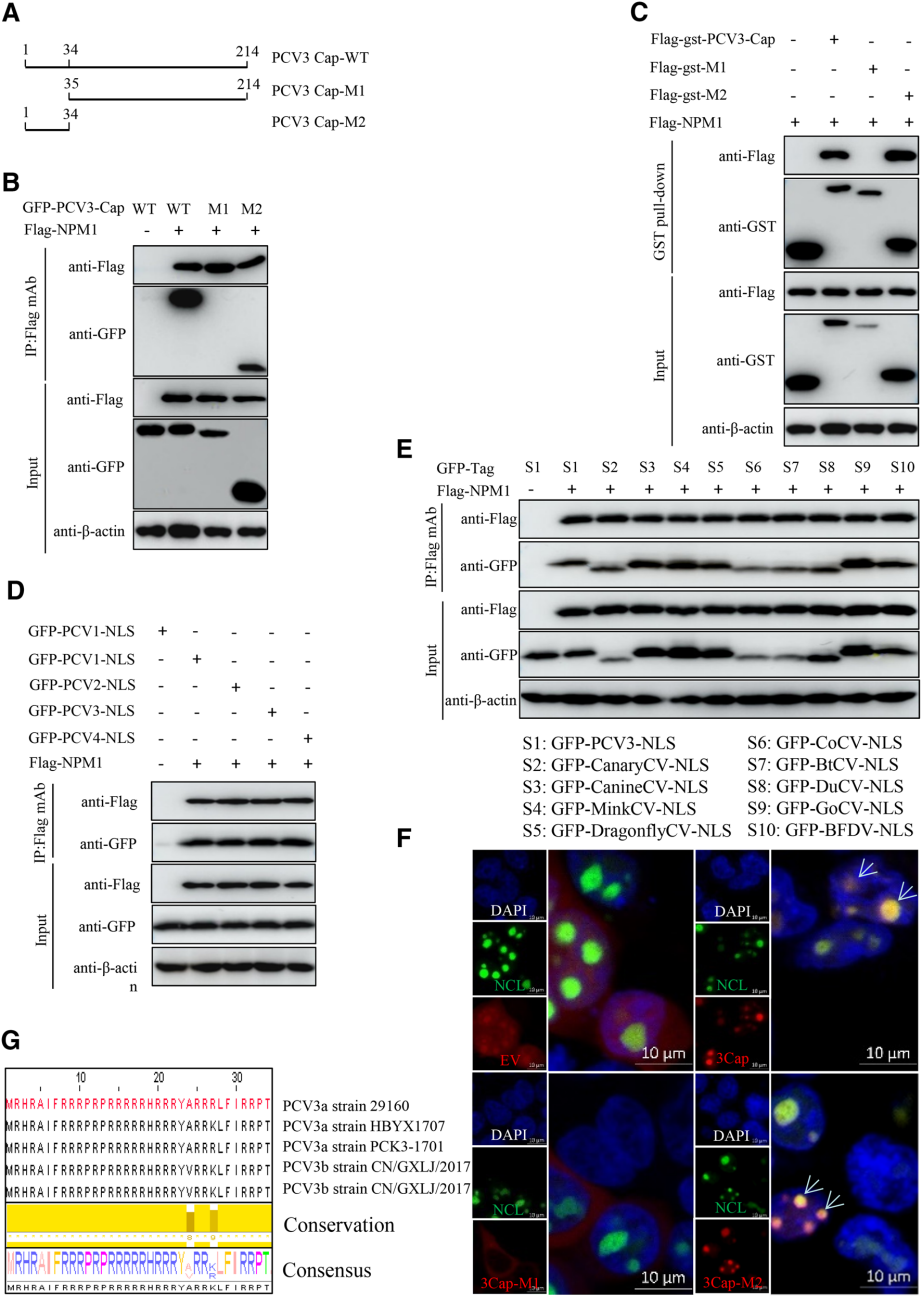
**Binding domain identification of PCV3 Cap with NPM1.** **A** Schematic representation of the truncation mutants of PCV3 Cap used in this study. **B**, **C** The N-terminal nuclear localization signal of PCV3 Cap interacted with NPM1. HEK293T cells were cotransfected with plasmids encoding full-length PCV3 Cap or truncation mutants fused with a GFP-, or Flag-gst tag, along with Flag-NPM1; cell lysates were subjected to immunoprecipitation or GST pull-down and immunoblotting using the indicated antibodies. **D**, **E** The nuclear localization signals (NLSs) within capsid protein of porcine circovirus type 1, 2, 3, 4 and circoviruses from other species were responsible for the binding to NPM1. HEK293T cells were cotransfected with plasmids encoding NLSs of PCV1, 2, 3, 4 (**D**) and circoviruses from terrestrial, aquatic and avian species, including pigs, canaries, canines, minks, dragonflies, pigeons, ducks, bats, geese, and parrots (**E**), along with Flag-NPM1; cell lysates were subjected to immunoprecipitation and immunoblotting using the indicated antibodies. **F** Identification of the nucleolar localization signal in PCV3 Cap. HEK293T cells were cotransfected with GFP-NCL and mCherry-PCV3-Cap-WT, -M1, or -M2 for 24 h. The resultant cells were fixed, stained with DAPI and subjected to confocal microscopy analysis. Scale bar, 10 μm. **G** Amino acid sequence alignment of the nucleolar localization signal from different PCV3 genotypes.

To identify the domain in PCV3 Cap required for interaction with NPM1, we constructed two truncated mutants of PCV3 Cap (Figure 3A). Co-IP and GST pull-down assays showed that amino acids (aa) 1–34 (M1), as well as the full-length PCV3 Cap (WT) could interact with NPM1, whereas aa 35–214 (M2) of Cap could not able to interact with NPM1 (Figure 3B, C). These data showed that the N-terminal residues 1–34 of PCV3 Cap that form an NLS (34) are crucial for Cap binding to NPM1. Further Co-IP experiments showed that the NLSs within capsid protein of porcine circovirus type 1, 2, 3, 4 and circoviruses from terrestrial, aquatic and avian species, including pigs, canaries, canines, minks, dragonflies, pigeons, ducks, bats, geese, and parrots, were also indispensable for the binding to NPM1 (Figure 3D, E), indicating that the interaction is highly conserved in evolution.

As shown in Figure 2A, B, PCV3 Cap is located in nucleolus in the transfected-cells. To investigate whether PCV3 Cap has a nucleolus localization signal (NoLS), we used online tools (NucleOlar location sequence Detector, http://www.compbio.dundee.ac.uk/www-nod/index.jsp; and NLS Mapper, http://nls-mapper.iab.keio.ac.jp/cgi-bin/NLS Mapper form.cgi). Surprisingly, a potential NoLS was predicted at the N-terminus of PCV3 Cap. To validate this, plasmids PCV3 Cap-WT, -M1, and -M2, were respectively cotransfected into 293T cells along with GFP-nucleolin (NCL). Confocal imaging showed that only PCV3 Cap-WT and -M2 colocalized with NCL (Figure 3F). These data confirmed that the N-terminal residues 1–34 of PCV3 Cap is a NoLS. Aligning the amino acid sequences of NLS from different genotypes (PCV3a, PCV3b) of PCV3 using Jalview software revealed that NLSs of PCV3 Cap are highly conserved (Figure 3G).

### Ser48 in OligoD of NPM1 is critical for binding to PCV3 Cap

**Figure 4:**
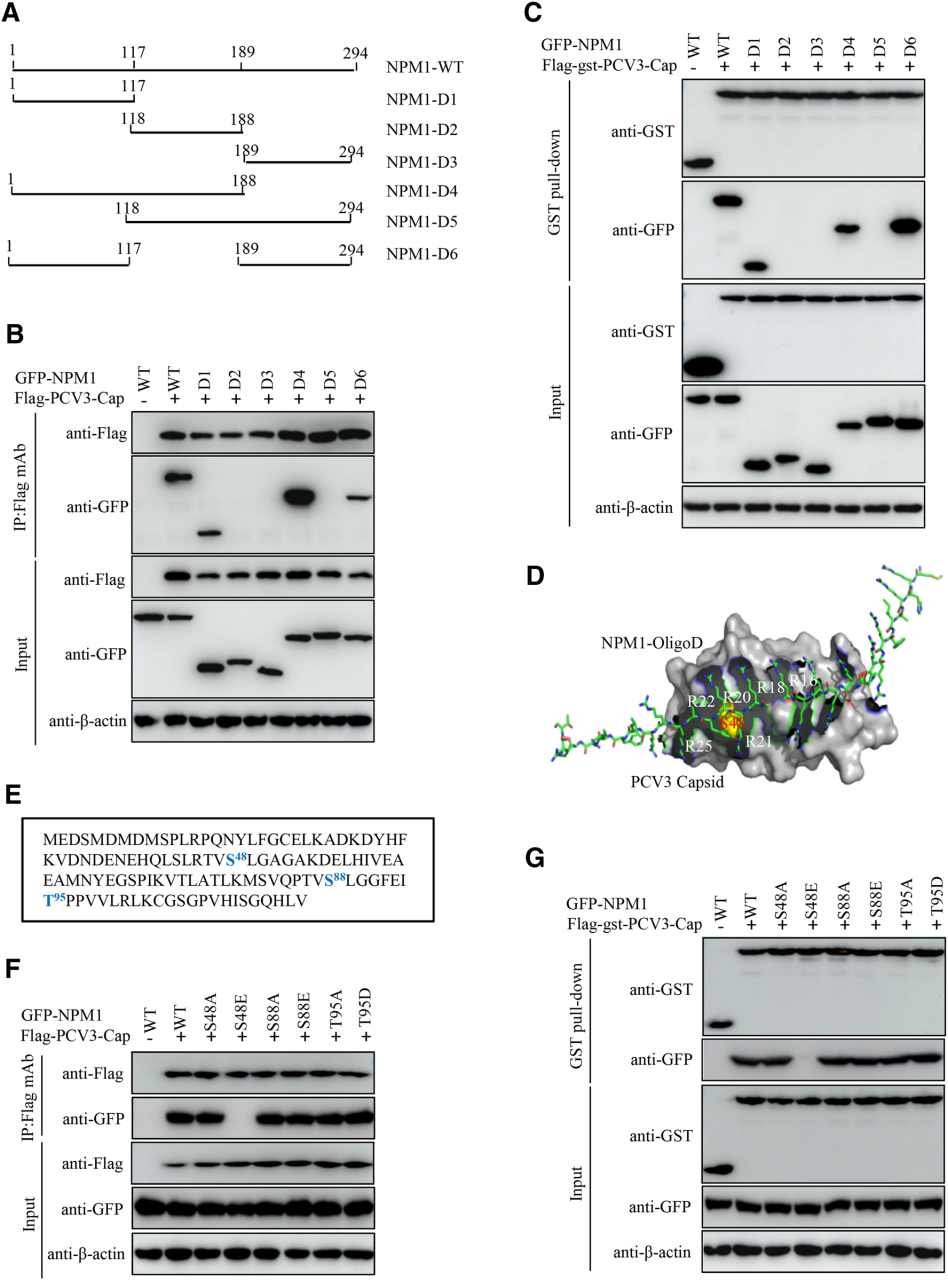
**Serine-48 of NPM1 is critical for binding to PCV3 Cap.** **A** Schematic representation of the OligoD, HistonD, and NBD of NPM1 and their truncation mutants used in this study. **B**, **C** The OligoD of NPM1 (aa 1–117) interacted with PCV3 Cap. HEK293T cells were cotransfected with plasmid GFP-NPM1-WT or serial GFP-NPM1 truncated mutants D1 to D6, along with Flag-PCV3-Cap and Flag-gst-PCV3-Cap expression vectors. The cell lysates were subjected to immunoprecipitation or GST pull-down and immunoblotting using the indicated antibodies. **D** Prediction of binding pattern of N-terminal OligoD from NPM1 with NoLS of PCV3 Cap using PyMOL software. **E** The N-terminal amino acid sequence of the OligoD of NPM1. Serine (S) or threonine (T) residues are marked in red. **F**, **G** Mapping the crucial amino acids of OligoD responsible for binding to PCV3 Cap. HEK293T cells were cotransfected with NPM1 or NPM1 mutants (-S48A, -S48E, -S88A, -S88E, -T95A, or -T95D), along with Flag-PCV3-Cap and Flag-gst-PCV3-Cap expression vectors, and the cell lysates were subjected to immunoprecipitation or GST pull-down and immunoblotting using the indicated antibodies.

Various functional domains have been identified within NPM1, including an N-terminal oligomerization domain (OligoD) having chaperone activity, the C-terminal nucleic acid binding domain (NBD), and two central acidic domains for histone binding (HistonD) [42]. To characterize the domain responsible for binding of NPM1 to PCV3 Cap, we constructed a series of NPM1-truncation mutants fused with GFP: OligoD (aa 1–117), HistonD (aa 118–188), NBD (aa 189–294), OligoD-HistonD (aa 1–188), HistonD-NBD (aa 118–294), OligoD-NBD (aa 1–117 + 189–294) (Figure 4A) and mapped the domains of NPM1 necessary for interaction with PCV3 Cap. Co-IP and GST pull-down assays showed that the constructs OligoD, OligoD-HistonD and OligoD-NBD interacted with PCV3 Cap, whereas HistonD, NBD and HistonD-NBD did not (Figure 4B, C), indicating that the OligoD of NPM1 is required for binding to PCV3 Cap. Binding pattern of the OligoD of NPM1 with PCV3 Cap NLS in silico using PyMOL software also showed that the N-terminal OligoD of NPM1 bound well to the NLS of PCV3 Cap in a plastic and flexible manner (Figure 4D).

Phosphorylation of the oligomerization domain is essential for the regulation of NPM1 structural polymorphism [47]. To explore if these phosphorylation sites played roles in interaction with PCV3 Cap and to identify the key amino acid residues in the OligoD critical for the interaction, we constructed a series of putative phosphorylation site mutants of GFP-NPM1, including GFP-NPM1-S(serine)48A(alanine), -S48E(glutamic acid), -S88A, -S88E, -T(threonine)95A, and -T95D(aspartic acid) (Figure 4E), and subjected to Co-IP and GST pull-down assays with Flag-PCV3-Cap and Flag-gst-PCV3-Cap. The results showed that the non-phosphorylated S48A, S88A and T95A, and mimic-phosphorylated S88E and T95D bound with Flag-PCV3-Cap and Flag-gst-PCV3-Cap nearly as well as the WT. However, the mimic-phosphorylated S48E mutant failed to binding to PCV3 Cap (Figure 4F, G). In summary, these findings indicate that the unphosphorylated serine-48 of NPM1 is responsible for the interaction between NPM1 and PCV3 Cap.

### Charge property of the 48th amino acid within NPM1 determines its nucleolar localization and NPM1/Cap interaction

**Figure 5:**
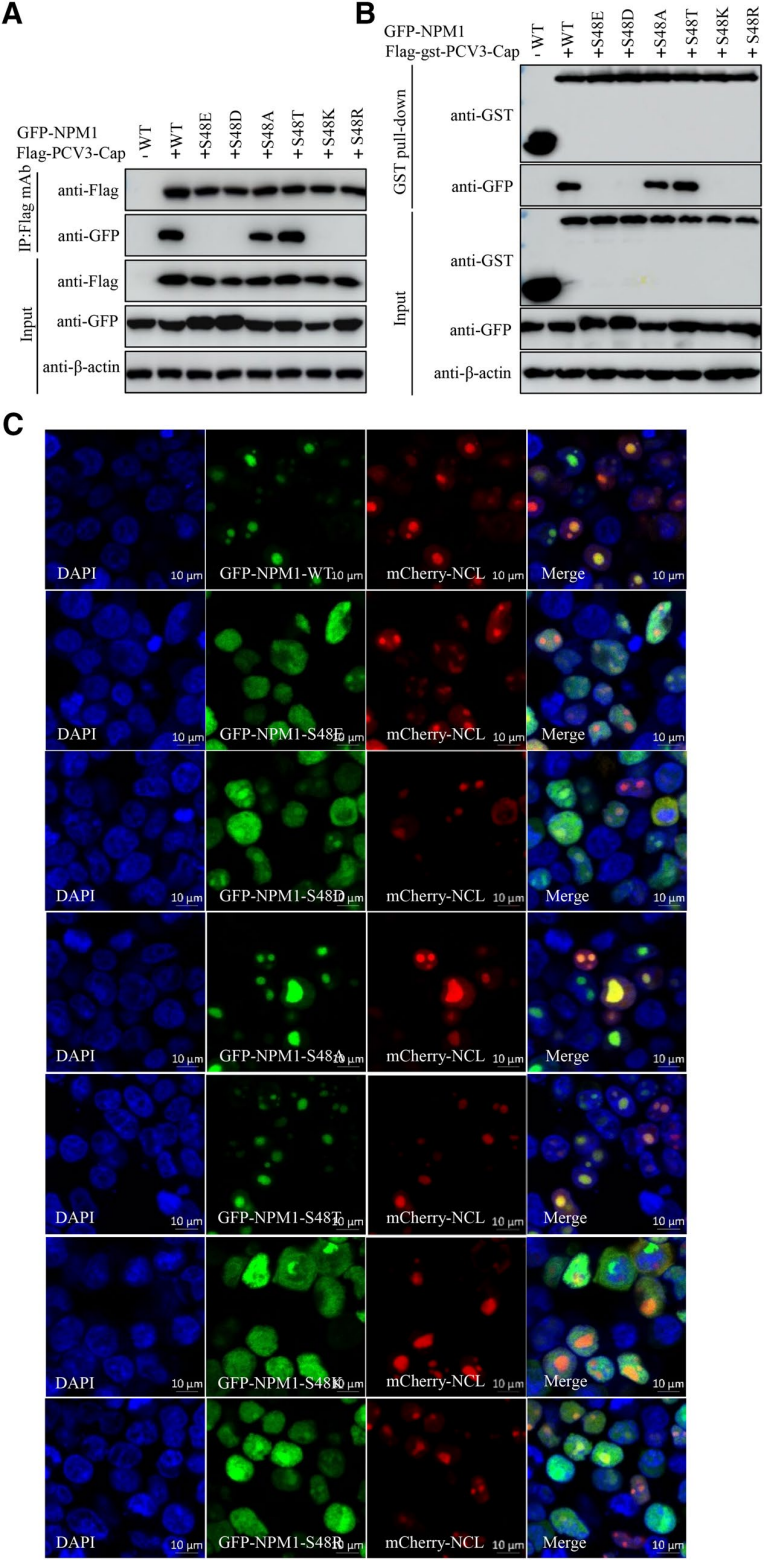
**Charge property of serine-48 within NPM1 determines its subcellular localization and interaction with PCV3 Cap.** **A**, **B** Charge property of serine-48 of NPM1 is responsible for binding to PCV3 Cap. HEK293T cells were cotransfected with NPM1 or NPM1 mutants (-S48E, -S48D, -S48A, -S48T, -S48K, or -S48R), along with Flag-PCV3-Cap and Flag-gst-PCV3-Cap expression vectors, and the cell lysates were subjected to immunoprecipitation or GST pull-down and immunoblotting using the indicated antibodies. **C** Nucleolin colocalization with NPM1 and various NPM1 mutants in transfected cells. HEK293T cells were cotransfected with GFP-NPM1 or NPM1 mutants (-S48E, -S48D, -S48A, -S48T, -S48K, or -S48R), along with mCherry-NCL for 24 h, and cells were fixed and then subjected to confocal microscopy analysis. Nuclei were stained with 4′, 6′-diamidino-2-phenylindole (DAPI). Scale bar, 10 μm.

The non-phosphorylated S48A, but not the mimic-phosphorylated S48E interacted with PCV3 Cap, which compelled us to further characterize the 48th amino acid. Due to the different charge properties of S48A and S48E, we investigated if charge property of 48^th^ amino acid within NPM1 is responsible for binding to PCV3 Cap. Overexpression plasmids of NPM1 with different charge property of 48th amino acid, including GFP-NPM1-WT, -S48E, -S48D, -S48A, -S48T, -S48K(lysine), and -S48R(arginine) were constructed and proceeded to Co-IP and GST pull-down assays with Flag-PCV3-Cap and Flag-gst-PCV3-Cap. The results showed that NPM1 harboring a neutral amino acid in position 48 retained interaction with PCV3 Cap, whereas switching the neutral amino acid to an acidic or basic one abolished this property (Figure 5A, B). Taken together, these data demonstrated that charge property of 48th amino acid in OligoD of NPM1 is critical for interaction with PCV3 Cap.

To further explore whether charge property of 48th amino acid within NPM1 made a difference in its nucleolar localization, we employed GFP fused NPM1 48th amino acid mutants (GFP-NPM1-WT, -S48E, -S48D, -S48A, -S48T, -S48K, -S48R) and nucleolus-located protein mCherry-NCL. Immunofluorescence assays showed that mutants S48A and S48T exhibited nucleolar localization and colocalized with NCL as same as WT, whereas the S48E, S48D, S48K, and S48R variants distributed into nucleoplasm uniformly, and failed to colocalize with NCL (Figure 5C), indicating that charge property of 48th amino acid within NPM1 is responsible for its nucleolar localization. Overall, we concluded that charge property of 48th amino acid within NPM1 plays crucial role in its nucleolar localization and interaction with PCV3 Cap.

## Discussion

NPM1 is a constitutively expressed protein which interacts with several host and viral proteins and participates in several steps of viral life cycle such as nuclear entry, viral genome replication, transcription, capsid assembly and egress [48–51]. Different domains of NPM1 enable this protein to shuttle between cytoplasm, nucleoplasm and nucleolus, but still, it primarily resides into the nucleolus. Under certain conditions, NPM1 may be transported to cytoplasm to perform specific functions, mediated by binding of chromosome region maintenance 1 (CRM1) to the nuclear export signal (NES) motifs or some other factors. However, in normal physiologic conditions, nuclear import predominates over export, and NPM1 is particularly resides in nucleolus to perform its functions [52].

As a multifunctional nucleolar phosphoprotein, NPM1 interacts with various viral proteins. Two stretches of negatively charged amino acids in the primary structure of NPM1 are particularly important because these motifs may potentially bind with positively-charged amino acid stretches in viral proteins, such as arginine-rich motif (ARM) and NLS [51]. Viral positively charged amino acid sequences always play important regulatory roles in different stages of the viral replication cycle, such as nucleocytoplasmic transport, viral genome replication and transcription, and viral particle assembly [50, 51]. However, whether PCV3 Cap, harboring a conserved NoLS, could also bind to NPM1 protein has not been reported. Our results showed that knockdown of *NPM1* suppressed the nucleolar localization of PCV3 Cap (Figure 1). PCV3 Cap colocalizes and interacts with NPM1 (Figure 2), and the NLSs within capsid protein of circoviruses from various species were all indispensable for binding to NPM1 (Figure 3), indicating that the interactions between NPM1 and circoviruses Cap NLSs are highly conserved during the evolution. Moreover, we verified that charge property of serine-48 within N-terminal oligomerization domain of NPM1 is vital for its subcellular localization and interaction with PCV3 Cap (Figures 4 and 5). PCV3 Cap may enter the nucleolus at the beginning of infection to support viral transcription, or alter the cell cycle with retention of the S phase and expression of related host proteins, or favor the synthetic replication of viral genome DNA as well [53]. During the infection cycle, the viral proteins Cap, Rep and Rep’ accumulate in the nucleoplasm, implying that DNA replication and encapsidation of the circular closed single-stranded DNA (ssDNA) occur in the nucleus and not in cytoplasmic compartments [53, 54]. The capsid assembly in the nucleus is a prerequisite for translocation to the cytoplasm and subsequent release of mature virions during late infection [55]. It will be interesting to investigate whether PCV3 Cap interacts with cellular factors regulating transcription.

Intriguingly, when Ser48 residue of NPM1 was mutated to neutral amino acids (Ala, Thr et al.), it retained nucleolar localization. But, when it was mutated to acidic amino acids (Glu, Asp) or alkaline amino acids (Lys, Arg, His), it did no longer localize in nucleolus (Figure 5). Phosphorylation of the oligomerization domain is responsible for the thermodynamic stability and spatial conformation of NPM1 and its nucleolar localization [47], we thus speculate that different charge properties of NPM1 affect its subcellular localization and interaction with other proteins. Viral exploitation of nucleolar function may lead to alterations in host cell transcription and translation, or to hijacking of nucleo–cytoplasmic transport and capsid assembly to favor viral replication [56]. Considering that the nucleolar localization signal (NoLS) at the N-terminus of PCV3 Cap functions as an NPM1 binding site and mediates its transport to the nucleolar compartment, we hypothesize that NPM1 promotes intracellular nucleolar trafficking of PCV3 Cap. The NPM1 targets PCV3 Cap to the nucleolus via interaction with its NoLS and facilitates encapsidation viral genome DNA, and assembly of viral particles and hence it is essential for viral replication inside the nucleus of infected cells, which is consistent with the previous study [57]. The NLS of PCV2 Cap can interact with the nuclear membrane receptor (gC1qR) to regulate DNA [58]. Likely, this suggests that the NLS of PCV3 Cap may be also involved in DNA binding. NPM1 may act as an adaptor for transport PCV3 Cap into nucleolus and facilitate its nucleolar localization. Further study is needed to verify whether the nuclear entry of PCV3 virion is also mediated by NPM1.

In summary, our results showed that knockdown of *NPM1* impaired the nucleolar localization of PCV3 Cap and charge property of 48th amino acid within NPM1 plays critical role in its nucleolar localization and interaction with PCV3 Cap. Taken together, this study broadens our understanding of nucleolar entry of PCV3 Cap and mechanism of PCV3 replication, which will be helpful to identify novel potential targets for therapeutic and prophylactic intervention of PCV3 infection.

## Data Availability

All data and materials generated for this study are included in the article.

## Abbreviations

NPM1: nucleolar phosphoprotein nucleophosmin-1
PCV: porcine circovirus
PCV1: porcine circovirus type 1
PCV2: porcine circovirus type 2
PCV3: porcine circovirus type 3
Cap: capsid protein
PCVAD: porcine circovirus-associated diseases
NoLS: nucleolar localization signal
NGS: next generation sequence
PDNS: porcine dermatitis and nephropathy syndrome
ORF: open reading frame
NLS: nuclear localization signal
STAT2: signal transducer and activator of transcription 2
HSV-1: herpes simplex virus type 1
AdV-2: adenovirus 2
IAV: influenza A virus
HCV: hepatitis C virus
NPC: nuclear pore complex
HSP70: heat shock protein 70
HDV: hepatitis delta virus
NCL: nucleolin
OligoD: oligomerization domain
HistonD: histone-binding domain
NBD: nucleic acid-binding domain
HIV-1: Human immunodeficiency virus type 1
MEM: minimal essential medium
DMEM: Dulbecco’s modified Eagle medium
FBS: fetal bovine serum
pAb: polyclonal antibody
mAb: monoclonal antibody
EGFP: enhanced green fluorescent protein
GST: glutathione S-transferase
HRP: horseradish peroxidase
PCR: polymerase chain reaction
CanaryCV: Canary circovirus
MinkCV: mink circovirus
DragonflyCV: dragonfly cyclovirus
CoCV: Columbid circovirus
BtCV: bat associated cyclovirus
DuCV: duck circovirus
GoCV: goose circovirus
BFDV: Beak and feather disease virus
PFA: paraformaldehyde
DAPI: 4′, 6′-Diamidino-2-phenylindole
SDS: sodium dodecyl sulfate
PAGE: polyacrylamide gel electrophoresis
PBS: phosphate-buffered saline
Co-IP: co-immunoprecipitation
RNAi: RNA interference
PI: propidium iodide
FACS: fluorescence-activated cell sorting
aa: amino acid
CRM1: chromosome region maintenance 1
NES: nuclear export signal
ARM: arginine-rich motif
ssDNA: single-stranded DNA
gC1qR: globular heads of complement component C1q receptor
